# The development of an end-to-end service solution to support lupus patients and improve their experience in clinical trials

**DOI:** 10.1177/1740774518811111

**Published:** 2018-11-14

**Authors:** Jodie Allen, Abbey Child, Sarah Mertens

**Affiliations:** 1 UCB Pharma, Slough, UK; 2 UCB Pharma, Brussels, Belgium

**Keywords:** Systemic lupus erythematosus, patient support program, recruitment, retention, adherence, clinical trials

## Abstract

**Objective:**

To develop an end-to-end clinical trial service to improve patient experience during trials, reduce the burden of participating in a trial, and increase trial retention.

**Methods:**

A literature search and stakeholder interviews were used to identify current challenges and unmet needs of systemic lupus erythematosus patients and other systemic lupus erythematosus clinical trial stakeholders. The results from the literature search and interviews were used to create a five-phase map describing the current clinical trial experience of all stakeholders. A set of proposed solutions were developed to address the identified unmet needs and challenges. These solutions were presented to trial-experienced patients and study site personnel; any feedback obtained was used to further refine the solutions.

**Results:**

Four site personnel and seven patients from three different systemic lupus erythematosus clinical trial sites were interviewed between September 2015 and December 2015. Key unmet needs and challenges were identified at each stage of the clinical trials. At the screening stage, some patients incorrectly thought they were successfully enrolled into the clinical trial. During enrollment, some patients found it difficult to keep fully informed about the trial and were unable to explain the trial process to loved ones. During the trial, patients struggled to prepare for study visits, felt overwhelmed by the trial process, and wanted someone to talk to for support. Clinical trial site personnel reported current key challenges as: delivering trial information clearly and consistently to patients, setting patient expectations, retaining enrolled patients, and providing non-clinical patient support. To address the needs of patients and site personnel, an end-to-end support service was designed, consisting of nine solutions: *My Best Choice, My Eligibility, My Lupus Trial Kit, My Lupus Trial Coach, My Appointment Guide, My Clinic Compass, Our Gratitude, Building a Different Network, and My Next Chapter*.

**Conclusion:**

The solutions proposed in this qualitative study may help improve the systemic lupus erythematosus clinical trial experience for patients, potentially helping to increase trial recruitment and retention. The solutions proposed here would also promote positive patient-trial personnel relationships, which may help site personnel identify patients at risk of early withdrawal, while ensuring that the time and resources of site personnel are used efficiently.

## Introduction

Clinical trials are necessary to evaluate the safety and efficacy of new interventions in the prevention and treatment of disease.^1^ For adequately powered clinical trials, large numbers of participants are often required.^2^ This can pose a challenge as factors such as the high number of appointments, inpatient hospital stays, and perceived discomfort from medical procedures can lead to poor recruitment and adherence, and high rates of patient drop-out.^1–5^ Poor participant recruitment and retention can result in clinical trial delays, which can lead to increased trial costs and increased risk of trial failure.^3^ Implementing small changes that reduce the trial burden faced by patients could help to increase participant numbers and minimize such risks.^3^

Systemic lupus erythematosus (SLE) is a chronic systemic autoimmune disease affecting multiple organs, including the kidneys and central nervous system.^6,7^ Symptoms include fever, fatigue, and arthralgia; cutaneous manifestations such as “butterfly rash” and skin lesions; joint swelling, oral ulcers, serositis, and irreversible alopecia.^6,7^ Changes in physical appearance, limitations in physical ability, muscle pain, and alopecia are associated with a number of psychological symptoms such as depression, anxiety, and mood disorders.^8^ Health-related quality-of-life scores in patients with SLE are typically lower than in the general population, with the greatest differences observed in the following domains: fatigue, applied cognition, pain interference, and Psychosocial Illness Impact-Negative (a Patient-Reported Outcomes Measurement Information System assessment instrument).^9^ Although earlier diagnosis and improved management have increased 5-year survival rates to 95%,^6^ SLE therapeutic options remain limited and are based on outcomes from only few randomized trials;^6,10^ in the last 50 years only one new drug has been approved.^11,12^

Many SLE trials have experienced difficulties in recruiting participants.^13^ For SLE patients, factors contributing to a patient’s decision to participate in SLE clinical trials include current health status, taking too many medications, time required to participate, disliking the idea of being randomized to placebo, and concerns that a medication will exacerbate their disease or cause side effects.^4,13^ To improve patient recruitment, recommendations have been developed to enhance the patient experience in clinical trials, based on input gathered from patients and patient representatives;^14^ these include improving communication and “customer care,” providing more opportunities for patients to ask questions, providing greater patient support, and improving logistics (i.e. travel to and around hospitals).^14^

The aim of this qualitative study was to develop an end-to-end clinical trial patient support service to help improve the patient’s experience during SLE clinical trials, to keep the burden of participating in a clinical trial as low as possible, and improve trial retention. The needs of SLE clinical trial patients and study personnel were determined from the literature and from interviews; support solutions were then co-created with trial-experienced patients and clinical trial site personnel. The solutions were intended to benefit all stakeholders involved in the conduct of a clinical trial: SLE patients and their families, site personnel and investigators, and trial sponsors.

## Methods

**Figure 1.**
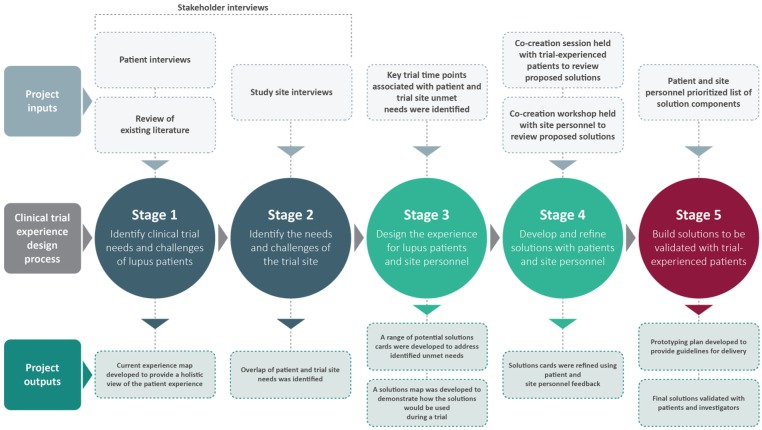
Overview of study design.

The needs of and challenges faced by patients and other stakeholders in SLE clinical trials were determined through a search of academic and gray literature, and via interviews conducted with stakeholders including patients, trial site personnel and sponsors. An overview of the study design is presented in Figure 1.

### Literature review

The aim of the literature search was to better understand the clinical trial experience from the patients’ perspective and identify the needs, context and challenges of patients, site personnel and sponsors involved in SLE clinical trials. The databases searched were PubMed and Google Scholar using search terms such as “clinical trial,” “experience,” “lupus,” “adherence,” “patient,” “concern,” “difficult,” “benefit,” “challenge,” and “care.” The search timeframe was 20 years, spanning from January 1997 to January 2017. Inclusion and exclusion criteria are presented in Table 1.

**Table 1.** Inclusion and exclusion criteria for articles, white papers, reports, and gray literature.

Inclusion criteria
Articles were available in English
The abstract contained information in relation to patient experience, either in conjunction with the clinical trial experience, or medication adherence
Articles related to patients with any chronic or non-communicable disease
Exclusion criteria
Articles were not available in English
Articles did not relate to clinical trial participation, or medication adherence
Articles related to communicable or acute disease

### Patient recruitment

Patients were recruited from six English-speaking clinical trial centers located in the United States. Trial sites were provided with a letter to send to clinical trial patients who had previously participated in an SLE clinical trial, inviting them to take part in an interview to discuss their experience of participating in the trial. All patients provided informed, written consent before joining the study.

### Site personnel identification

Clinical trial personnel at the same six clinical trial sites were invited by letter to participate in an informal interview about their experience of caring for patients in the context of a recent SLE clinical trial. Personnel from three trial sites participated in the process. To maintain anonymity, no demographic information was collected from site personnel participating in the study. There were no other inclusion or exclusion criteria.

### Interview methodology

UCB Pharma conducted all patient interviews by telephone. An independent market research agency conducted site personnel interviews. All interviews were audio recorded for data analysis purposes. Interviews were conducted using thematic interview guides, containing specific questions for patients (Supplementary Table 1) or site personnel (Supplementary Table 2). Interview transcripts were analyzed using thematic analysis to map the patient experience to specific clinical trial time points, as described below.

### Experience mapping

Content from the literature search and qualitative interviews was combined to create a 5-phase map describing the current clinical experience. The five clinical trial phases included learning about the trial, screening, trial participation, toward the end of the trial, and the end of the trial. Overlapping themes identified during the thematic analysis were grouped together. A research coordinator, a senior clinical trial nurse, and the principal investigator at one of the participating study sites (Emory University and Grady Memorial Hospital, Atlanta, US) reviewed the experience map to ensure that all site needs and challenges had been captured. The finalized experience map was used to guide the development of a set of proposed solutions to improve the end-to-end patient experience during an SLE clinical trial.

### End-to-end patient support service design

Potential solutions that addressed the unmet needs and challenges identified during the experience mapping exercise were created using a design thinking approach, a design methodology that provides a solution-based approach to solving problems.^15^ Potential solutions were prioritized based on desirability, viability, and feasibility (i.e. the ease of implementation).

#### Patient co-creation sessions

Patients were invited to attend an individual co-creation session at the Emory University and Grady Memorial Hospital, in March 2016. During the co-creation session, the proposed solutions were presented to the trial-experienced patient. The co-creation sessions were run by an external agency and aimed to elicit patient feedback on the design of the end-to-end patient support solutions.

#### Site personnel co-creation session

The proposed solutions were also presented to study site personnel in a group co-creation session held in March 2016 at the Emory University and Grady Memorial Hospital. The group co-creation session was conducted by the same external agency, with the same aim of eliciting feedback on the proposed design of the end-to-end patient support solution.

Feedback from the co-creation sessions was used to design a “solutions map.” The map was used to assess the delivery timings and interactions between different solutions to ensure they formed a coherent end-to-end service. The solutions were refined using feedback from the co-creation sessions with patients and site personnel, and an implementation manual was developed; this ensured that the end-to-end experience was implemented as intended, that is, the right patient receives the right solution, at the right time, and delivered in the right way. A subsample of four patients and three site personnel was presented with the final solutions to ensure the solutions were in line with the needs they had expressed.

### Subject anonymity and confidentiality

Site personnel interviews were conducted by an independent market research agency on an opt-in basis, enabling anonymity of all participating site personnel. Co-creation sessions were conducted by an independent service design agency. Patients were interviewed by UCB Pharma (preventing anonymity) but all interview data were treated as confidential and stored on secured networks and drives. For the co-creation sessions, patient names and telephone numbers were sent to UCB Pharma using an encrypted file transfer service. Consent forms were used for participating patients. All data recorded remained confidential.

### Ethical approval

UCB compliance and ethics processes were followed for all interactions with stakeholders, in addition to governance processes followed by the participating sites. All participants provided written informed consent before participating in the study. As the study was a service evaluation study, institutional board review (IRB) approval was not required.

## Results

### Patient interview demographics

All patients who took part were female and SLE disease duration ranged from 2 to 16 years. Of the six clinical trial sites contacted, three sites sent letters to all patients who had taken part in an SLE clinical trial. The minimum and maximum time that patients had participated in an SLE trial was 1 day and 4.7 years (244 weeks), respectively. Of the 20 patients identified, 11 patients responded to the invitation letter and 6 out of the 11 patients completed the interview. The demographics of patients who participated in the interviews are presented in Table 2.

**Table 2.** Demographics of patients participating in the interview and co-creation session.

	Interview n (%)	Co-creation session n (%)
Age group		
18–29	0 (0)	2 (13)
30–39	2 (33)	8 (53)
40–59	3 (50)	4 (27)
≥60	1 (17)	1 (7)
Ethnic group		
Asian	0 (0)	Not available
Black or African American	2 (33)	Not available
White	4 (67)	Not available
Other/Mixed	0 (0)	Not available

### Stakeholder interviews and co-creation sessions

A total of 10 stakeholders were interviewed (4 site personnel and 6 patients). Semi-structured interviews conducted over the telephone took place between September 2015 and December 2015. An additional 20 trial-experienced patients were invited to take part the one-to-one co-creation session; 17 patients agreed to take part, 15 patients participated in the co-creation session (2 patients were lost to follow-up). The demographics of patients who participated in the co-creation session are presented in Table 2.

### Current and desired patient experience

**Figure 2.**
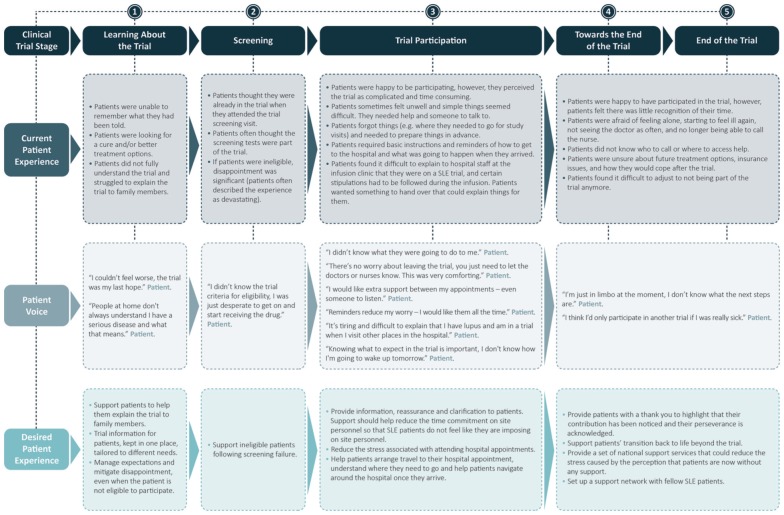
Current and desired patient experience.

A map describing the current patient experience of participating in a clinical trial and the desired patient experience is presented in Figure 2. At the screening stage, some patients incorrectly thought they were already in the trial because, as part of the screening procedure, patients had to sign consent forms and undergo assessments to confirm their eligibility. A few patients who subsequently found they were ineligible reported significant disappointment. Keeping patients fully informed about the clinical trial was identified as a key unmet need in the literature review and patient interviews. Patients were often unable to capture and retain all information, and found it difficult to accurately explain the clinical trial process to their family and friends. Furthermore, patients had a tendency to forget things, perhaps as a result of the Lupus-related cognitive dysfunction which is commonly known to SLE patients as “Lupus fog.”^16^ As a result of this, patients often required help explaining the benefits of the trial to family and friends who frequently viewed their participation as an unnecessary risk to the patient’s health, and their ability to work and care for their family. As the majority of symptoms were not visible, friends and family often underestimated the impact of SLE symptoms on the patient’s life.

Patients struggled to prepare for their study visits and found it difficult to navigate around a large hospital. Patients also wanted to have someone to talk during the trial, and felt overwhelmed by the trial process and the clinic, requiring basic instructions and regular reminders about what would happen and when, during each study visit. Although patients were happy to have participated in a clinical trial, their SLE symptoms often meant they felt very unwell; consequently, patients were often desperate to take part in the trial as they thought the trial drug would alleviate the symptoms of their disease. Their desperation may be compounded by the fact that only one new drug has been approved for SLE treatment in the last 50 years.^11,12^ Toward the end of the trial, patients required help and support readjusting to life outside of the trial setting, something that clinical trial personnel struggled to routinely provide. Patients also felt that the study sponsor did not provide sufficient recognition of their time and investment in the trial.

### Site needs

**Figure 3.**
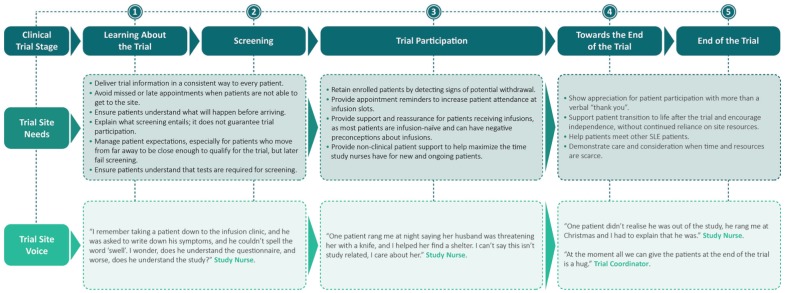
Site needs. SLE: systemic lupus erythematosus.

The specific needs of site personnel identified during their interviews are presented in Figure 3. Key challenges reported included delivering trial information in a clear and consistent way to every patient, setting patient expectations during the screening procedure, retaining enrolled patients, and the need to keep reminding patients of the time of their appointments and how to prepare for them. Site personnel reported that the study coordinator or nurse often became the patients’“go-to” person for non-trial-related support with topics as wide ranging as insurance, housing, disability, and legal paperwork. At the end of the trial, site personnel reported that they received little training on how to support patients with their transition away from the trial, despite a need to encourage patient independence to allow more time for site personnel to care for new and ongoing patients.

### Lupus clinical trial solutions

**Figure 4.**
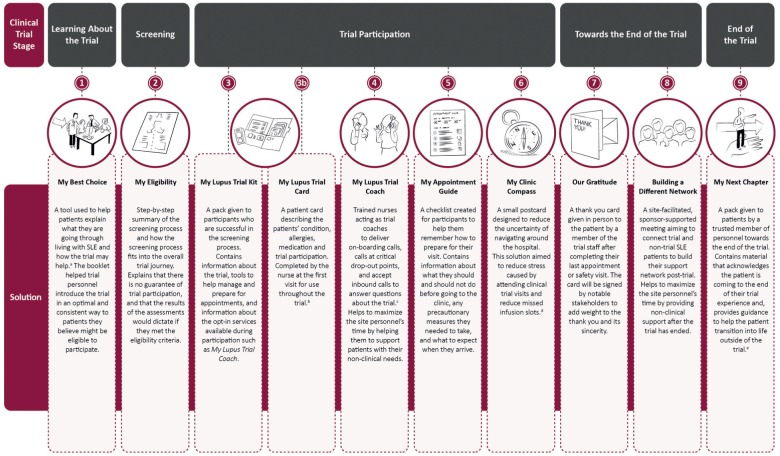
Lupus clinical trial experience solutions. ^a^*My Best Choice* includes information on SLE, the trial objectives, and the safety measures in place; ^b^*My Lupus Trial Card* allows patients to assert themselves using the right information at the right time in a busy hospital; ^c^*My Lupus Trial Coach* accepts inbound calls to answer patients’ questions about the trial while addressing their emotional needs and respecting the protocol; ^d^*My Clinic Compass* has form-fillable spaces that patients can fill in when meeting the study nurse for the first time; ^e^*My Next Chapter* also outlines the wider picture of the trial by illustrating the number of patients and countries that are participating. SLE: systemic lupus erythematosus.

Following the co-creation sessions, a set of SLE clinical trial solutions were developed and reviewed by trial-experienced patients and site personnel. Their feedback was used to iteratively improve the proposed solutions. To address the needs of patients and site personnel, and to improve the end-to-end clinical trial experience for SLE patients, nine solutions were developed (Figure 4). The solutions identified as key are described in detail below.

The first solution designed to address patient and site needs is *My Best Choice*, a printed booklet designed to be introduced to the patient by the clinical trial physician at the beginning of the trial, prior to enrollment. Patients would then be encouraged to take the booklet home to read and discuss the content with their families. To help enhance family engagement, a clinical trial physician could also deliver the content of the booklet via video. Using lay language, *My Best Choice* will present key information about the seriousness and variability of SLE symptoms and communicate the trial objectives, safety measures and support available to patients taking part in the trial, helping to support the patient during the decision-making process. It will also help patients explain to their family and friends what they were going through living with the disease, and help to provide reassurance that participation in the trial is a well-considered option, for which safety measures are in place and that they would be closely monitored. This solution will also help personnel members introduce the trial to patients in a consistent way.

*My Eligibility* is a one-page printed card with a diagram demonstrating the two possible outcomes, trial eligibility and ineligibility, to be introduced to the patient by the physician alongside *My Best Choice.* To qualify for screening, some patients had moved their families and left their jobs, something they were eager to do for the opportunity to try a new treatment. Consequently, being ineligible to take part in the trial led to significant patient disappointment. *My Eligibility* provides a step-by-step summary of the screening process, explains why specific tests will be undertaken, and how the screening process fits into the overall trial journey. It is designed to provide better information about the screening process, manage expectations, and mitigate the patients’ disappointment if they are ineligible to take part. *My Eligibility* aims to manage patients’ expectations by clearly explaining that screening is not a guarantee to participate in the trial, and that the results of the screening will dictate if they meet the entry requirements. This solution helps site personnel better explain the screening process and eligibility to the patient.

*My Lupus Trial Coach* is an opt-in support solution provided by specialist nurses trained to have a good understanding of the study protocol and adverse event reporting. The nurse takes on the role of trial coach, supporting the patient from the start until the end of the clinical trial. The overall aim of this solution is to improve patient enrollment and retention and was designed to be introduced by the principal investigator or the lead study nurse to instigate trust and ensure patient uptake. The trial coach delivers on-boarding phone calls to patients during the early weeks of the trial, and at critical points associated with patient drop-out. The trial coach will receive inbound calls to answer any questions about the trial, or their participation, that the patient may have previously been reluctant to ask the busy trial nurse. *My Lupus Trial Coach* is intended to make patients feel supported throughout the trial by providing a coach they could call for any trial-related queries or worries. This would ensure that site personnel, who typically provide this support to patients, have more time available to focus on trial-related activities without having to provide additional out-of-hours support to patients.

*Building a Different Network* is a site-facilitated, sponsor-supported, informal meeting where all personnel, patients, and family members can share their experiences of the trial and living with SLE. The meeting will connect trial and non-trial SLE patients as the trial is coming to the end. This solution provides another level of patient support and will help patients access information about future treatment options; this information is particularly necessary as the medication patients will receive as part of the trial will no longer be available to them. *Building a Different Network* is designed to support patients’ transition back to being non-trial SLE patients. Patients described being a “normal” patient as receiving less care and no longer having a dedicated support team looking after them. This solution aims to connect SLE patients as the trial nears the end and encourages patient independence to free up the time of site personnel who often continue to provide non-clinical support after the trials have finished.

*My Next Chapter.* At the end of the *Building a Different Network* meeting, patients will be provided with the final support solution, named *My Next Chapter*. This is a support pack containing an information booklet and a personalized thank you card signed by the study sponsor and trial site personnel. Patients often felt there was little recognition for their time and investment in the trial and *My Next Chapter* recognizes patients’ contributions. Patients reported the need for help transitioning to life after the trial had finished. Patients who were underinsured, or uninsured, felt this would mean less, or no care. *My Next Chapter* acknowledges that patients are coming to the end of their trial experience. It helps patients transition back to life outside of the clinical trial environment by providing guidance and information about what they need to do once the trial is over, how to manage their illness, and who to talk to if they have any questions or concerns.

## Discussion

The clinical trial experience can negatively affect patient recruitment, enrollment, and adherence and lead to high rates of patient drop-out.^1–5^ Poor recruitment results in delays in over 81% of clinical trials,^3^ and this leads to significantly higher costs and slower clinical development.^3,17^ In some cases, clinical trials have been discontinued due to low retention.^4^ The aim of this qualitative study was to develop an end-to-end clinical trial patient support service to help improve the patient experience during clinical trials, thereby improving retention. Interviews and a literature search were used to assess the needs of patients and study personnel involved in SLE clinical trials, culminating in the development of an SLE clinical trial end-to-end service, consisting of nine solutions. All solutions were developed in collaboration with trial-experienced patients and study personnel.

This qualitative study identified patient education and the provision of clear instructions at the start of the trial as key unmet needs. A study conducted by Bevan et al. (1993) reported a similar finding; 60% of clinical trial patients would have liked written information about the clinical trial at the start of the trial, however, only 38% received it. *My Best Choice, My Eligibility*, and *My Lupus Trial Kit* are solutions designed to fulfill this need.^18^ In a study by Lim et al.,^19^ two simulations of draft phase 2 and 3 anifrolumab studies in SLE and lupus nephritis (LN) patients were conducted to assess patient concerns and preferences during the clinical trial and to identify factors that may improve clinical study protocols and conduct. The clinical trial simulations involved four phases: site feasibility assessment, patient recruitment, simulation of two clinical trial visits, and a debrief session.^19^ The authors reported that participating patients found background material valuable, preferred knowledgeable site personnel, and appreciated support from their family and friends.^19^ Consistent with the findings of our qualitative study, Lim et al.^19^ recommended that patients should be provided with information about their disease and the trial, that family and friends should also be educated about the participants’ disease and the clinical trial, and that SLE patients should be encouraged to engage with each other.

A patient support program developed for patients with chronic inflammatory disease receiving adalimumab provides access to support solution components and was associated with increased treatment adherence.^20^ The solution components included face-to-face interactions with a registered nurse ambassador, financial assistance, phone, text and email patient reminders, a supply of training resources, and follow-up calls from a nurse to maintain patient contact.^20^ Together with the findings of our study, these results highlight the importance of end-to-end solutions with content tailored to the disease-specific needs of the trial participants. A systematic literature review of patient support programs used in chronic diseases provided further evidence that patient support programs have a positive impact on adherence, clinical, and humanistic outcomes.^21^ These studies highlight the value of providing patient support programs, and further support our proposal that the end-to-end service solution designed in this study would improve the clinical trial experience for patients.

Placebo effects can be caused by factors that are distinct from either the therapeutics or placebo controls used in a clinical trial and can impact the results of a trial if not carefully controlled. For example, improvements to patients’ symptoms through clinical trial participation, including emotional engagement with clinicians, can enhance the effect of the study drug.^22^ Clinical trials are often conducted worldwide across multiple clinical trial centers and so it is difficult to control all factors contributing to the placebo effect. The end-to-end service solution proposed in this study may help introduce consistency in the patient experience across clinical trial centers and may help to mitigate placebo-related effects; however, this is yet to be determined.

To ensure the safety and well-being of clinical trial participants, and the integrity of data collected, every interaction point with a patient should be carefully planned in the clinical trial protocol.^23^ The protocols describe how the trial should be run and so create an opportunity to integrate the patient experience as part of the clinical trial. Currently, patient information leaflets are provided as part of the informed consent procedure. Patients value these information sources as they help with the decision to participate in a trial, improve understanding about the trial, reduce decisional conflict, and lessen any regret associated with taking part in a clinical trial.^24,25^ However, participants do not always fully understand the rationale for conducting the trial or processes involved when considering participation or once they have enrolled.^24^ Including solutions, such as those proposed in this study, as part of the protocol would ensure all patients enrolled in the trial receive the same information at the correct time; this “patient experience protocol” would guarantee that all patients receive an optimal trial experience and would enable full alignment of all study centers running the trial.

This study was associated with several limitations. The proposed end-to-end service solution was developed and face-validated using patient and stakeholder interviews only; although the end-to-end solution was based on recent clinical trial experience, no formal validation was used to test the solution in a clinical trial setting prior to publication. The socioeconomic status and education level of the patients were not considered when designing the solutions and the same patients and personnel involved in the development of the solutions, reviewed the final version. Therefore, it would be important to test the final solutions on an independent group of site personnel and patients. Finally, although patients were recruited from previous SLE trials, the study involved 21 patients whose experiences were not intended to be fully generalizable to the wider SLE patient population. SLE disproportionally affects certain racial groups.^26–28^ Therefore, the end-to-end service solution may need to be adapted to ensure the materials provided are culturally sensitive and have the greatest potential impact on different patient groups. The end-to-end solutions were designed to address the needs of SLE patients enrolled into a clinical trial and so require minimal modification or customization.

In conclusion, the end-to-end service solution proposed in this study has the potential to improve the SLE clinical trial experience for both patients and site personnel by improving patient on-boarding, and providing a dedicated support service for patients, which may help site personnel to spend more of their time caring for patients. This will provide site personnel with more time to focus on their designated healthcare tasks, which will in turn improve the overall patient experience. The solution proposed here would also ensure positive patient-trial personnel relationships develop from the start of the trial, which may help site personnel identify patients at risk of early withdrawal, potentially reducing the number of patient drop-outs.

## Supplemental Material

811111_suup_mat_1 – Supplemental material for The development of an end-to-end service solution to support lupus patients and improve their experience in clinical trials Click here for additional data file. Supplemental material, 811111_suup_mat_1 for The development of an end-to-end service solution to support lupus patients and improve their experience in clinical trials by Jodie Allen, Abbey Child and Sarah Mertens in Clinical Trials

## References

[1] The National Academies Press. The prevention and treatment of missing data in clinical trials 2010. Available at: https://www.nap.edu/read/12955/chapter/1 (accessed December 2017). 24983040

[2] Prescott RJ Counsell CE Gillespie WJ et al Factors that limit the quality, number and progress of randomised controlled trials. Health Technol Assess 1999; 3: 1–143. 10683591

[3] Choi CB Bae SC Gupta S et al Improving participation in clinical trials of novel therapies: going back to basics. Rheum Dis Clin North Am 2014; 40: 553–559, ix. 10.1016/j.rdc.2014.05.002 25034162

[4] Costenbader KH Karlson EW Gall V et al Barriers to a trial of atherosclerosis prevention in systemic lupus erythematosus. Arthritis Rheum 2005; 53: 718–723. 16208639 10.1002/art.21441

[5] Sully BG Julious SA Nicholl J. A reinvestigation of recruitment to randomised, controlled, multicenter trials: a review of trials funded by two UK funding agencies. Trials 2013; 14: 166. 23758961 10.1186/1745-6215-14-166 PMC3691846

[6] Kuhn A Bonsmann G Anders HJ et al The diagnosis and treatment of systemic lupus erythematosus. Dtsch Arztebl Int 2015; 112: 423–432. 26179016 10.3238/arztebl.2015.0423 PMC4558874

[7] Tsokos GC Gordon C Smolen JS. Systemic lupus erythematosus. 1st ed Philadelphia, PA: Mosby, Elsevier, 2007.

[8] Beckerman NL Auerbach C Blanco I. Psychosocial dimensions of SLE: implications for the health care team. J Multidiscip Healthc 2011; 4: 63–72. 21594059 10.2147/JMDH.S19303 PMC3093952

[9] Lai JS Beaumont JL Jensen SE et al An evaluation of health-related quality of life in patients with systemic lupus erythematosus using PROMIS and Neuro-QoL. Clin Rheumatol 2017; 36: 555–562. 27848056 10.1007/s10067-016-3476-6

[10] La Paglia GMC Leone MC Lepri G et al One year in review 2017: systemic lupus erythematosus. Clin Exp Rheumatol 2017; 35: 551–561. 28721860

[11] European Medicines Agency. Benlysta EPAR summary for the public. Available at: http://www.ema.europa.eu/docs/en_GB/document_library/EPAR_-_Summary_for_the_public/human/002015/WC500110153.pdf (accessed December 2017).

[12] Benlysta. About Benlysta. Available at: https://www.benlysta.com/about/index.html (accessed February 2018).

[13] Costenbader KH Brome D Blanch D et al Factors determining participation in prevention trials among systemic lupus erythematosus patients: a qualitative study. Arthritis Rheum 2007; 57: 49–55. 17266094 10.1002/art.22480

[14] Eli Lilly and Company. Improving the patient experience of clinical trials. Report no. UKLLG00017, April 2014.

[15] Ideo. Design thinking. Available at: https://www.ideou.com/pages/design-thinking (accessed November 2017).

[16] Lupus Foundation of America. National resource center on lupus. Available at: https://resources.lupus.org/entry/how-lupus-affects-memory (accessed December 2017).

[17] Kitterman DR Cheng SK Dilts DM et al The prevalence and economic impact of low-enrolling clinical studies at an academic medical center. Acad Med 2011; 86: 1360–1366. 21952064 10.1097/ACM.0b013e3182306440 PMC3203249

[18] Bevan EG Chee LC McGhee SM et al Patients’ attitudes to participation in clinical trials. Br J Clin Pharmacol 1993; 35: 204–207. 8443040 PMC1381516

[19] Lim SS Kivitz AJ McKinnell D et al Simulating clinical trial visits yields patient insights into study design and recruitment. Patient Prefer Adherence 2017; 11: 1295–1307. 28814837 10.2147/PPA.S137416 PMC5545635

[20] Rubin DT Mittal M Davis M et al Impact of a patient support program on patient adherence to adalimumab and direct medical costs in Crohn’s disease, ulcerative colitis, rheumatoid arthritis, psoriasis, psoriatic arthritis, and ankylosing spondylitis. J Manag Care Spec Pharm 2017; 23: 859–867. 28737994 10.18553/jmcp.2017.16272 PMC10397981

[21] Ganguli A Clewell J Shillington AC. The impact of patient support programs on adherence, clinical, humanistic, and economic patient outcomes: a targeted systematic review. Patient Prefer Adherence 2016; 10: 711–725. 27175071 10.2147/PPA.S101175 PMC4854257

[22] Kaptchuk TJ Miller FG. Placebo effects in medicine. N Engl J Med 2015; 373: 8–9. 26132938 10.1056/NEJMp1504023

[23] University of California San Francisco. Clinical research resource HUB- clinical trial protocol development. Available at: https://hub.ucsf.edu/protocol-development (accessed November 2017).

[24] Gillies K Skea ZC Campbell MK. Decision aids for randomised controlled trials: a qualitative exploration of stakeholders’ views. BMJ Open 2014; 4: e005734. 10.1136/bmjopen-2014-005734 PMC4139633 25138811

[25] Juraskova I Butow P Bonner C et al Improving decision making about clinical trial participation—a randomised controlled trial of a decision aid for women considering participation in the IBIS-II breast cancer prevention trial. Br J Cancer 2014; 111: 1–7. 24892447 10.1038/bjc.2014.144 PMC4090720

[26] Lim SS Bayakly AR Helmick CG et al The incidence and prevalence of systemic lupus erythematosus, 2002–2004: the Georgia lupus registry. Arthritis Rheumatol 2014; 66: 357–368. 24504808 10.1002/art.38239 PMC4617771

[27] Rees F Doherty M Grainge M et al The incidence and prevalence of systemic lupus erythematosus in the UK, 1999–2012. Ann Rheum Dis 2016; 75: 136–141. 25265938 10.1136/annrheumdis-2014-206334 PMC4717400

[28] Somers EC Marder W Cagnoli P et al Population-based incidence and prevalence of systemic lupus erythematosus: the Michigan lupus epidemiology and surveillance program. Arthritis Rheumatol 2014; 66: 369–378. 24504809 10.1002/art.38238 PMC4198147

