# Supplementary material for: The development of an end-to-end service solution to support lupus patients and improve their experience in clinical trials
Source: Clin Trials. 2018 Nov 14;16(1):71–80. doi: 10.1177/1740774518811111 (PMC6364094; doi:10.1177/1740774518811111)
Supplement: 811111_suup_mat_1 – Supplemental material for The development of an end-to-end service solution to support lupus patients and improve their experience in clinical trials [file 811111_suup_mat_1.pdf]

The Development of an End-to-End Service Solution to Support Lupus Patients and Improve their Experience in Clinical Trials

Jodie Allen[1], Abbey Child[1] and Sarah Mertens[2]

[1]UCB Pharma, Slough, United Kingdom; [2]UCB Pharma, Brussels, Belgium.

Correspondence to: Jodie Allen, UCB Pharma, 208 Bath Rd, Slough, SL1 3WE. Email: Jodie.Allen@ucb.com. Telephone number: +44 7768 107597

SUPPLEMENTARY MATERIALS

SUPPLEMENTARY DATA

Supplementary Table 1. Patient interview questions

Patient Interview Questions

Introduction

1. Can you start by telling me a little about yourself?

Before the Clinical Trial

1. Thinking back to when you joined the trial, what was going on in your life at the time?
2. What did you know about clinical trials before? Had you taken part in any other forms of research? If so, can you tell me about this?
3. How did you come to learn of this clinical trial? What were your initial thoughts or feelings?
4. What information were you given when you decided whether to take part or not?
5. What information or person might have been helpful to you when deciding?
6. What were you told about the trial? What did you understand to be the benefits of taking part?
7. Were you worried about anything?
8. Who did you talk to about the trial? Did you speak with anyone else?
9. When you were deciding whether to participate did you talk about it with anyone else, at home or with peers, for example? What kind of things did you talk about?
10. What would you say influenced your decision to take part the most?
11. What information were you given (e.g. leaflets)? What do you remember about these, if anything?

At Enrolment in the Clinical Trial

1. Could you describe what you saw when you first visited the clinic / the infusion room / assessment room. What about lighting? What did the chairs look like? What were people doing? What were people wearing (clothing), what was on the walls? Any pictures? What could you see? What noises did you hear?
2. Can you tell me about joining the trial? What was it like?
3. What did you understand would happen during the trial?
4. Was anything a surprise during the trial?
5. What questions can you remember asking during the trial? What things did you want to know? Can you tell me about this?
6. Did you have a main contact person? What kinds of things did you talk with them about?

During the Clinical Trial

1. Can you walk me through one of your visits step by step?
2. Could you describe what you saw when you first saw the infusion room / treatment / assessment room? What about lighting? What did the chairs look like? What were people doing? What were people wearing (clothing), what was on the walls? Any pictures? What could you see? What noises did you hear?
3. Did anyone ask you about your experience while participating? If yes, can you tell me about this? If no, what do you wish they had asked you?
4. Can you tell me about how you travelled to your visits?
5. Can you tell me about what you were normally feeling the day before your visits? What were you feeling on the morning of your visit?
6. Did anyone come with you to your visits? Can you tell me about that?
7. How did the visits fit into your everyday life?
8. How could we have made it easier for you to attend?
9. How did you feel during the infusion (visits?) How could we have improved this time for you?
10. How would you describe the [lead physician/ your rheumatologist] in three words?
11. How would you describe the [perfect physician/rheumatologist] in three words? Can you tell me more about [safe], what did they do that made you feel [safe]?
12. Did anything happen that you did not expect? Did anyone tell you anything that you didn't expect to be told?
13. Can you tell me about what was going on in your life during the trial? [prompt about life events AND illness (flairs etc.)]
14. Can you remember one conversation that you had with a loved one during the trial? Can you tell me about this conversation?

After the Clinical Trial

1. How did you feel when the trial was coming to an end?
2. What did you expect to happen after the trial?
3. What happened during your final visit to the rheumatologist, what would you have liked to discuss, if anything?
4. Is there anything the trial staff could have done for you after the trial had finished?
5. What happens to the information collected during the trial?
6. What were you told about the success of the trial?
7. If someone were about to join this clinical trial, what advice would you give them?
8. If you could go back in time, would you still participate in this trial? Can you tell me more?

Supplementary Table 2. Site personnel interview questions

Site Personnel Interview Questions

Your role

1. Can you start by telling me a little about your role?
2. And briefly, what is your involvement with the recent lupus trial?

Trial Motivations

1. In your experience, what motivated participants to take part in this trial?
2. Other than the trial criteria, are there particular types of patients who are more likely to participate than others? What types of things make people more or less likely to take part?
3. Again, other than trial criteria, are there barriers to participation for some people? What are these?
4. What do you consider to be the benefits to patients of taking part in the trial? What do patients get from the experience?
5. To what extent do you think patients understand the benefits of taking part?

Trial Understanding and Expectations

1. Before they join, how would you describe patients' understanding and expectations of clinical trials?
2. How do patients' expectations match with the experience that they have?
3. What types of concerns do patients tend to have?

On-Boarding

1. Can you talk me through the 'joining' experience for patients, from an initial enquiry about taking part up to them joining?
2. What conversations are had with HCPs / study coordinators?
3. What information is provided? How – in person, by phone, on paper?
4. What do you think this part of the experience is like from a patient's point of view?
5. What kinds of questions do patients ask most frequently?
6. What is the strangest question you have been asked by a patient?
7. What do you think of the a) materials, b) collateral and c) website?
8. What is this part of the experience like from your point of view as a professional?
9. (If not provided) What, if any, are the challenges you face as a professional?

Visits

1. Can you talk me through a typical screening visit for this trial?
    a. Where does this take place?
    b. Who does the patient come into contact with?
    c. What happens?
    d. Who comes with the patient?
    e. What do you think the experience is like from a patient's point of view?
    f. What is this part of the experience like from your point of view as a professional?
    g. Can you tell me an example of a screening visit that did not go to plan?
2. **Can you talk me through a typical infusion visit for this trial?**
    a. Where does this take place?
    b. Who does the patient come into contact with?
    c. What happens?
    d. Who comes with the patient?
    e. What do you think the experience is like from a patient's point of view?
    f. What do patients talk about / do during these visits?
    g. What is this part of the experience like from your point of view as a professional?

Last Few Months of the Trial

1. Can you talk me through the last trial visit?
2. Can you talk me through the typical ways a trial might come to an end for a patient?
    a. What happens?
    b. Who is involved?
    c. What do you think the experience is like from a patient's point of view?
    d. What kinds of questions do patients ask in the last few months of a trial? Do certain patients ask certain questions?
    e. What is this part of the experience like from your point of view as a professional?
    f. What if anything, do you find challenging in the last few months of a trial?

Partners Involved in Delivering the Trial

1. Can you talk me through the organizations and people involved in making a trial like this happen?
2. What are the key challenges in delivering a trial of this nature from your professional perspective?
3. With the trial taking place in many locations around the world, how much variation do you think there is in patients' experiences? What factors might make the experience different and how?

Your View on Improving Patient Experiences

1. If you could start from a blank page, and money was no object, how would you design this trial? What would you change?
